# Novel Targeted Anti-Tumor Nanoparticles Developed from Folic Acid-Modified 2-Deoxyglucose

**DOI:** 10.3390/ijms20030697

**Published:** 2019-02-06

**Authors:** Shaoming Jin, Zhongyao Du, Huiyuan Guo, Hao Zhang, Fazheng Ren, Pengjie Wang

**Affiliations:** 1 Beijing Advanced Innovation Center for Food Nutrition and Human Health, College of Food Science & Nutritional Engineering, China Agricultural University, Beijing 100083, China; myjackyming@126.com (S.J.); duzhongyao@cau.edu.cn (Z.D.); renfazheng@cau.edu.cn (F.R.); 2 National Institutes for Food and Drug Control, Beijing 100050, China; 3 Beijing Laboratory of Food Quality and Safety, Beijing Higher Institution Engineering Research Center of Animal Product, Beijing 100083, China; guohuiyuan99@gmail.com (H.G.); zhanghaocau@cau.edu.cn (H.Z.)

**Keywords:** folic acid, 2-Deoxyglucose, nano-particles, targeted anti-tumor, self-assemble

## Abstract

The glucose analog, 2-deoxyglucose (2-DG), specifically inhibits glycolysis of cancer cells and interferes with the growth of cancer cells. However, the excellent water solubility of 2-DG makes it difficult to be concentrated in tumor cells. In this study, a targeted nano-pharmacosome was developed with folic acid-modified 2-DG (FA-2-DG) by using amino ethanol as a cleavable linker. FA-2-DG was able to self-assemble, forming nano-particles with diameters of 10–30 nm. The biological effects were evaluated with cell viability assays and flow cytometry analysis. Compared with a physical mixture of folic acid and 2-DG, FA-2-DG clearly reduced cell viability and resulted in cell cycle arrest. A computational study involving docking simulation suggested that FA-2-DG can dock into the same receptor as folic acid, thus confirming that the structural modification did not affect the targeting performance. The results indicated that the nano-pharmacosome consisting of FA-2-DG can be used for targeting in a nano-drug delivery system.

## 1. Introduction

Cancer has become the disease with the second highest mortality rate worldwide. Currently, the main therapeutic method for cancer is chemotherapy. However, chemotherapy in cancer treatment is usually accompanied by adverse effects, since many drugs cannot differentiate between cancerous cells and normal cells. The targeting of active substances to the tumor region is an effective measure to solve this problem.

Blocking the consumption of glucose in cancer cells has been shown to be an effective anti-tumor therapy [1,2,3], because the up-regulation of glycolysis is a near-universal trait in cancer cells that results in perturbed energy metabolism in cancer cells, in both normoxic and hypoxic environments [4,5,6]. Thus, inhibiting the glucose transporters on the cell membrane and the glycolytic enzymes in the cytoplasm has great potential for killing cancer cells.

The 2-Deoxyglucose (2-DG) is a structural analogue of glucose whose structural difference lies mainly in the hydroxyl group on the second carbon atom. The 2-DG inhibits glycolysis and reverses cancer cell metastasis [7,8,9,10]. It can inhibit the glucose transporters (GLUTs) on the membranes of cancer cells because it enters the cytoplasm in the same manner as glucose, via GLUTs [11]. After entering the cell, 2-DG is phosphorylated by hexokinase, the first key enzyme in glycolysis, forming2-DG-6-phosphate (2-DG-6-P). The 2-DG-6-P is a substrate of phosphoglucose isomerase, the second key enzyme in glycolysis, but it cannot be further catalyzed into fructose-6-phosphate (F-6-P). Thus, the process of glycolysis can be efficiently blocked. The inhibition of hexokinase is noncompetitive, but the inhibition of phosphoglucose isomerase is competitive [12]. Owing to these inhibitions, the metabolism of glucose is disturbed, resulting in a decreased energy supply, cell cycle arrest and inhibited cell viability [13].

However, the excellent water solubility of 2-DG makes it widely distributed in the body. Therefore, to increase its concentration in tumor cells, targeted transportation of 2-DG to tumors is a necessary strategy. Highly expressed receptors on the surfaces of cancer cells are a potential target for anti-cancer therapy [14,15]. The folate receptor, one of these highly expressed receptors, is widely used in anti-tumor targeting research [16,17,18]. Folic acid, the ligand of the folate receptor, is broadly used in the research and development of targeted anti-tumor drugs. It can be labeled or loaded with polymers, quantum dots and nano-materials, for targeting drug delivery. The forms of these nano-scale drug delivery systems vary considerably, from large polymers with polyethylene glycols [19] to moderate compositions with metals or quantum dots [20,21] to small nanoparticles with antineoplastic drugs [22,23]. Regardless of the form of the delivery system, the objective of improving efficacy and reducing toxicity can be achieved, confirming the advantages of nano-scale drug delivery systems [24,25].

Herein, we designed and synthesized a new drug delivery system consisting of folic acid-modified 2-DG (FA-2-DG). The synthesized small molecule is amphipathic and can self-assemble into nanoparticles and achieve both passive targeting and active targeting roles. Its biological activity was evaluated on the basis of cytotoxic effects in vitro in MCF-7 and SKOV-3 tumor cells, and the related mechanism of action was assessed by evaluating its influence on the cell cycle and its ability to induce apoptosis.

## 2. Results and Discussion

### 2.1. The Synthesis of FA-2-DG

**Figure 1:**
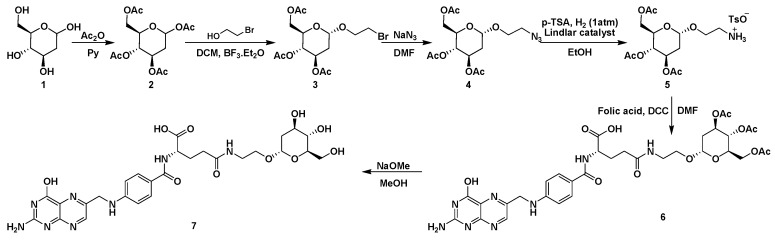
The synthesis route of FA-2-DG. Ac_2_O: acetic anhydride, Py: pyridine, DCM: dichloromethane, DMF: dimethylformamide, DCC: dicyclohexylcarbodiimide.

FA-2-DG was synthesized from folic acid and 2-DG, with amino ethanol as a linker agent. Since the high reaction activity of amino group would introduce by-products and make purification more difficult, amino ethanol was not directly used in synthetic reactions. First, 1-bromo ethanol was connected to acetylated 2-DG, and then the bromine atom was replaced by an azide group. In the next step, an amino group was generated via a reduction reaction catalyzed by a Lindlar catalyst under a hydrogen atmosphere. The amino and carboxyl groups formed an amide bond. After removal of the acetyl protection of 2-DG under alkaline conditions, FA-2-DG was synthesized. All structures were confirmed by 1H-NMR and 13C-NMR. The synthetic route is shown in Figure 1.

### 2.2. Characterization of the Nano-Properties

To evaluate the self-assembly performance of FA-2-DG, we characterized its nano-properties. Three different methods were applied: dynamic light scattering (DLS), transmission electron microscopy (TEM) and atomic force microscopy (AFM).

**Figure 2:**
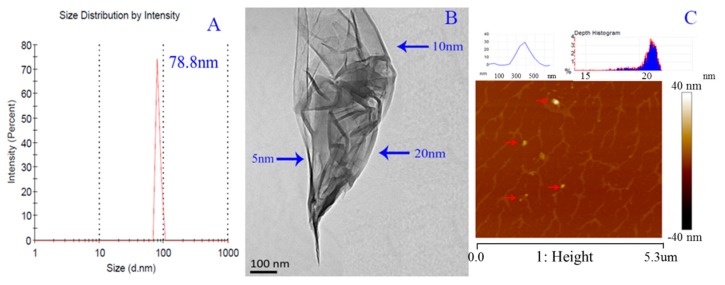
Nano-properties of FA-2-DG, (**A**) DLS size analysis of FA-2-DG in water solution; (**B**) TEM micrograph of FA-2-DG; (**C**) AFM image of FA-2-DG.

The DLS results showed that the peak diameter of FA-2-DG nano-particles in water was approximately 78.8 nm, as shown in Figure 2A. Only one narrow peak in the graph was observed, thus indicating that the particles were fairly homogeneous. The TEM results showed that the FA-2-DG self-assembled into nano-fibers, as shown in Figure 2B. The nano-fibers further assembled into nanoclusters. The positions shown by the blue arrow in Figure 2B indicate that the nano-fibers had diameters ranging from 5 nm to 20 nm, indicating the assembly process of continuous aggregates from fine to coarse. The AFM scans displayed the three-dimensional features of the sample, as shown in Figure 2C. FA-2-DG also formed nano-fibers and at the ends of the fibers (the positions indicated by the red arrows in Figure 2C), nano-particles with sizes of 20–30 nm were also found. The upper-left part of Figure 2C shows that the depth of particles was around 30 nm, and the upper-right part of the figure shows a particle diameter of around 25 nm. By plotting the absorbance of a set of FA-2-DG solutions as a function of FA-2-DG concentration, the critical aggregation concentration of FA-2-DG was 7.94 × 10^−3^ mM.

From all the three characterizations, the particle morphology and self-assembly capability of FA-2-DG was confirmed, and the nano-scale assembly results were validated. The DLS results showed the highest particle size, and the AFM results showed the largest particle size; the reason for this result was the differences in sample states. The TEM and AFM results were consistent: both images showed nano-fibers self-assembled from FA-2-DG.

### 2.3. Cell Viability Assays

**Figure 3:**
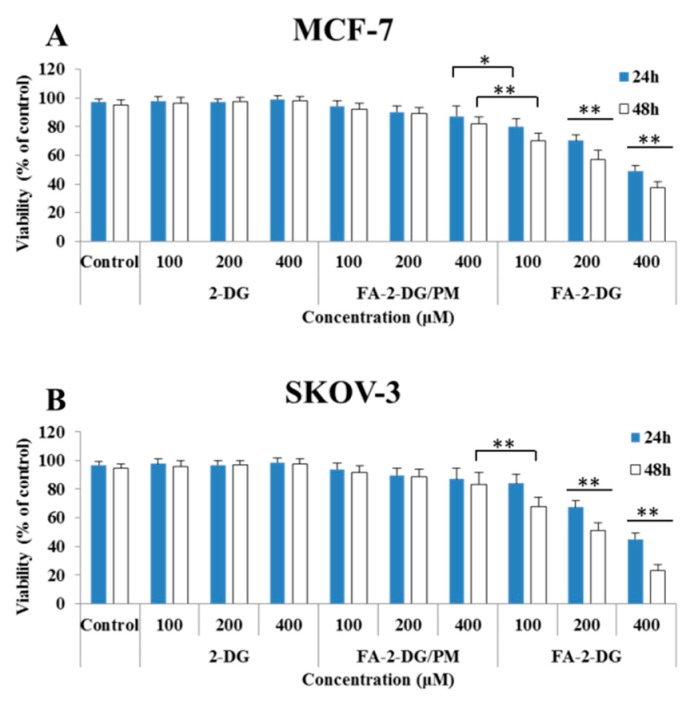
Cell viability assays of different treatments. (**A**) MCF-7 cells were treated with different groups of compounds for 24 h and 48 h. (**B**) SKOV-3 cells were treated with different groups of compounds for 24 h and 48 h. Cell viability was determined by MTT assays. The results are expressed as a mean percentage of controls ± standard deviation (SD) in three separate experiments. Significance was calculated by ANOVA and Duncan’s test (* *p* < 0.05, ** *p* < 0.01).

In vitro 3-(4,5-Dimethyl-2-thiazolyl)-2,5-diphenyl-2*H*-tetrazolium bromide (MTT) cytotoxicity assays were used to evaluate the biological effects of FA-2-DG on cell viability. In addition to a FA-2-DG and the blank control group, a third group treated with a physical mixture of folic acid and 2-DG (FA-2-DG/PM) was also evaluated. The results in Figure 3 showed that, compared with 2-DG, FA-2-DG/PM caused a modest decrease in cell viability at the high concentration of 400 μM, but FA-2-DG clearly inhibited cell viability in both MCF-7 and SKOV-3 cells. The inhibition of FA-2-DG/PM may have resulted from the weak hydrogen bond between folic acid and 2-DG. With increased time and concentration, the inhibition of FA-2-DG in both cell lines increased. On one hand, the nanoparticles showed advantages in anti-tumor treatment, but on the other hand, the results confirmed that the target of action was to interfere with glycolysis, and a long time was necessary to show better results. For the two cell lines, FA-2-DG showed better (no statistical analysis) inhibition than did SKOV-3 cells, because SKOV-3 cells expressed higher levels of folate receptors. Therefore, the SKOV-3 cell line was used for further flow cytometric analysis. Because 2-DG did not influence cell viability at the tested concentrations, it was unnecessary to analyze the effects on the cell cycle and cell apoptosis.

### 2.4. Effects of FA-2-DG on the Cell Cycle in SKOV-3 Cells

**Figure 4:**
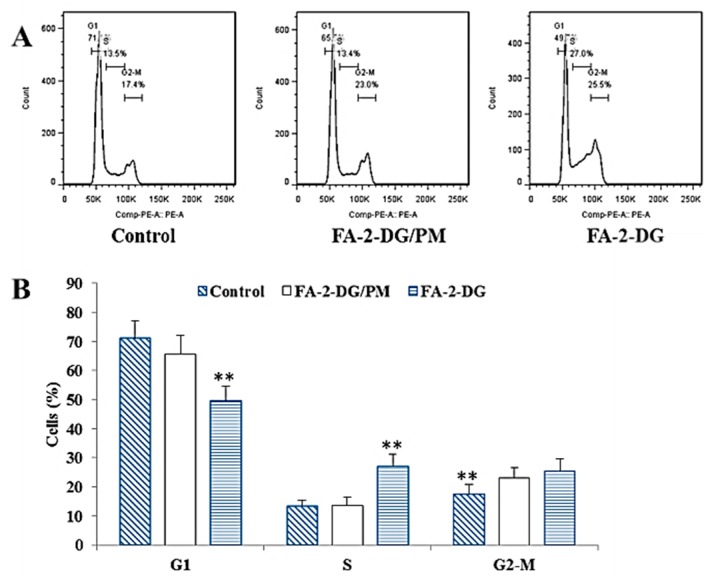
The effect of FA-2-DG on cell cycle regulation in SKOV-3 cells. (**A**) The cells were treated with the blank control and the other two groups of compounds at concentrations of 400 μM for 24 h, stained with propidium iodide (PI) and then subjected to flow cytometry analysis to determine the cell distribution at each phase of the cell cycle. The representative results from three independent experiments are shown. (**B**) The results are expressed as means ± SD of three independent experiments. Significant difference from the control was determined by using Student’s *t*-test (** *p* < 0.01).

To investigate whether FA-2-DG affected the cell cycle distribution of SKOV-3 cells, we treated the cells with different groups of compounds for 24 h and then analyzed cell cycle progression using flow cytometry. As shown in Figure 4A, FA-2-DG showed clear blocking of the cell cycle, and the frequency of S-phase cells increased significantly. Figure 4B showed the proportion in each cell cycle after treatment with three different groups. These results provide strong evidence of interference with energy metabolism, ineffective DNA replication due to decreased ATP production, and more cells arrested in S-phase.

### 2.5. Effects of FA-2-DG on the Induction of Apoptosis in SKOV-3 Cells

**Figure 5:**
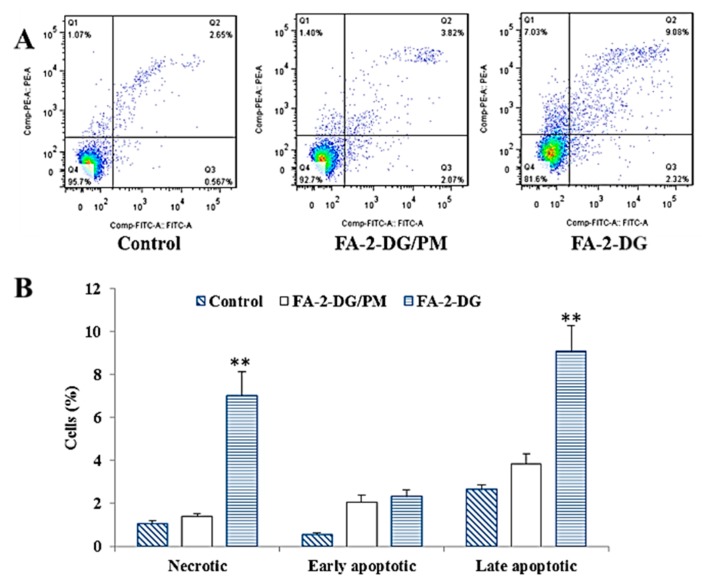
The effect of FA-2-DG on apoptosis in SKOV-3 cells. (**A**) The cells were treated with the blank control and the other two groups of compounds at concentrations of 400 μM for 48 h, then stained with Annexin V and PI. (**A**) The effect of FA-2-DG on cell death was determined via Annexin V-FITC/PI analysis by using flow cytometry. The representative results from three independent experiments are shown. (**B**) The results are expressed as means ± SD of three independent experiments. Significant difference from the control was determined by using Student’s *t*-test (** *p* < 0.01).

To further confirm that the FA-2-DG induced cell death was apoptosis, we performed flow cytometry, using Annexin V and PI staining. As shown in Figure 5A, the ratio of late apoptotic cells (upper right quadrant, Annexin V/PI positive) increased from 2.65% to 9.08% after 48 h of exposure to 400 μM of FA-2-DG. Figure 5B showed the proportion of cells in different apoptotic stages after three different treatments. Although the result of 9.08% did not provide clear evidence that FA-2-DG induced apoptosis, compared with FA-2-DG/PM, the effect was statistically highly significant.

### 2.6. Docking Studies

**Figure 6:**
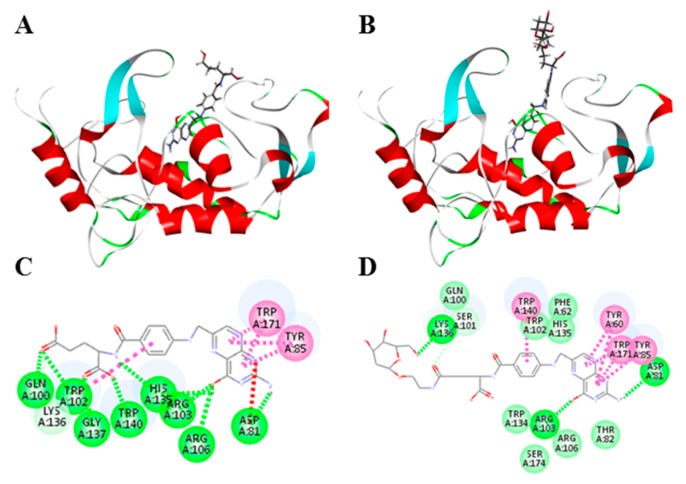
Docking studies of FA-2-DG with the folate receptor. (**A**) Folic acid docking into the folate receptor, red color parts represents the alpha helix in the protein structure, light blue color parts represents the beta folding; (**B**) FA-2-DG docking into the folate receptor, red color parts represents the alpha helix in the protein structure, light blue color parts represents the beta folding; (**C**) the 2-D diagram of ligand–receptor interactions between folic acid and the folate receptor; (**D**) 2-D diagram of ligand–receptor interactions between FA-2-DG and the folate receptor.

Docking studies were performed to verify that FA-2-DG targets folate receptors. As positive controls, a folic acid and folate receptor (PDB ID:4LRH) docking model was used with preferred docking orientation, as shown in Figure 6A; the cdocker energy was −59.5 Kcal·mol^−1^, and the interaction energy was −61.5 Kcal·mol^−1^. FA-2-DG docked into the interaction sphere in the same manner as folic acid, as shown in Figure 6B; the cdocker energy was −56.5 Kcal·mol^−1^, and the interaction energy was −59.5 Kcal·mol^−1^. To further understand the binding mode of FA-2-DG to the folate receptor, we performed a careful inspection of the docking pose in Figure 6C,D. In addition to folic acid and receptor interactions, FA-2-DG showed more interactions with amino acids than folic acid in the receptor, such as TYR60, SER101 and TRP134, suggesting that FA-2-DG can target the folate receptor in a more firm way than folic acid.

## 3. Materials and Methods

### 3.1. Materials

Folic acid and 2-DG were purchased from Sigma-Aldrich (St. Louis, MO, USA). Other chemicals, solvents and biochemical reagents were of analytical grade and were purchased from commercial sources (J&K Scientific, Beijing, China; Solarbio Science & Technology, Beijing, China).

### 3.2. Preparation of FA-2-DG

#### 3.2.1. Preparation of (4*R*,5*S*,6*R*)-6-(acetoxymethyl) tetrahydro-2*H*-pyran-2,4,5-triyl triacetate (compound **2**)

A solution of 5.0 g (30.5 mmol) of 2-DG (compound **1**) and 25 mL pyridine was prepared. To that solution, 23.8 g (233 mmol) of acetic anhydride was added dropwise at 0 °C. The reaction mixture was stirred at 25 °C for 16 h. After evaporation, the residue was diluted with 100 mL ethyl acetate. The organic layers were washed with citric acid (30 mL × 3), NaHCO_3_ (30 mL × 3) and brine (30 mL × 3), and then dried with anhydrous Na_2_SO_4_. After filtration, the filtrate was evaporated, and the residue was purified by column chromatography (SiO_2_, petroleum ether/ethyl acetate = 10/1 to 5/1) to obtain acetyl 2-DG (compound **2** shown in synthetic route in Figure 1, 9.1 g, 27.39 mmol, 89.9% yield) as a colorless oil. ^1^H-NMR (400 MHz, CDCl_3_): δ/ppm = 5.77 (dd, *J* = 10.0, 2.20 Hz, 1H), 5.08–4.97 (m, 2H), 4.31–4.26 (m, 1H), 4.01–4.03 (m, 1H), 3.74–3.70 (m, 1H), 2.35–2.29 (m, 1H), 2.09–1.90 (m, 12H). ^13^C-NMR (100 MHz, CDCl_3_): δ/ppm = 170.71, 170.10, 169.76, 168.79, 91.08, 72.87, 70.15, 68.26, 61.95, 34.73, 20.96, 20.86, 20.77, 20.71. HRMS (*m*/*z*): calcd for C_14_H_20_O_9_ [M + Na]^+^: 355.0999, found: 355.1008.

#### 3.2.2. Preparation of (2*R*,3*S*,4*R*)-2-(acetoxymethyl)-6-(2-bromoethoxy) Tetrahydro-2*H*-Pyran-3,4-diyl Diacetate (Compound **3**)

To a solution of compound **2** (5.0 g, 15 mmol), 2-bromoethanol (2.3 g, 18 mmol, 1.3 mL) in dichloromethane (35 mL) was added dropwise to BF_3_.Et_2_O (2.6 g, 18 mmol, 2.2 mL) at 0 °C. After stirring at 25 °C for 2 h, the reaction mixture was diluted with ice saturated NaHCO_3_ water solution (60 mL) and extracted with dichloromethane (50 mL × 2). The combined organic layers were washed with brine (40 mL × 3), then dried over anhydrous Na_2_SO_4_. After filtration, the filtrate was evaporated, and the residue was purified by column chromatography (SiO_2_, petroleum ether/ethyl acetate = 10/1 to 5/1) to obtain compound **3** (4.6 g, 10.4 mmol, 69.3% yield, 90.0% purity) as a yellow oil [26]. ^1^H-NMR (400 MHz, CDCl_3_): δ/ppm = 5.33–5.26 (m, 1H), 5.01–4.95 (m, 2H), 4.28–4.24 (m, 1H), 4.08–4.04 (m, 2H), 3.94–3.88 (m, 1H), 3.82–3.76 (m, 1H),3.48 (t, *J* = 0.8 Hz, 2H), 2.29–2.25 (m, 1H), 2.11–2.98 (m, 9H), 1.85–1.76 (m, 1H). ^13^C-NMR (100 MHz, CDCl_3_): δ/ppm = 170.71, 170.18, 169.93, 97.25, 69.24, 68.91, 68.31, 67.97, 62.33, 34.88, 30.09, 20.98, 20.78, 20.75. HRMS (*m*/*z*): calcd for C_14_H_21_BrO_8_ [M + Na]^+^: 419.0312, found: 419.0324.

#### 3.2.3. Preparation of (2*R*,3*S*,4*R*)-2-(acetoxymethyl)-6-(2-azidoethoxy) tetrahydro-2*H*-pyran-3,4-diyl Diacetate (Compound **4**)

Compound **3** (4.5 g, 11.3 mmol) and NaN_3_ (1.11 g, 16.9 mmol) in DMF (35 mL) were heated at 60 °C for 16 h. The mixture was diluted with ice water (300 mL) and extracted with ethyl acetate (100 mL × 2). The combined organic layers were washed with brine (40 mL × 3), dried over anhydrous Na_2_SO_4_, filtered and concentrated under reduced pressure to obtain (2*R*,3*S*,4*R*)-2-(acetoxymethyl)-6-(2-azidoethoxy) tetrahydro-2*H*-pyran-3,4-diyl diacetate (3.9 g, crude) as a yellow oil [27]. ^1^H-NMR (400 MHz, CDCl_3_): δ/ppm = 5.23–5.21 (m, 1H), 4.92 (brs 2H), 4.23–4.19 (m, 1H), 3.99–3.92 (m, 2H), 3.77–3.73 (m, 1H), 3.60–3.51 (m, 1H), 3.36–3.35 (m, 2H), 2.23–2.19 (m, 1H), 2.01–2.91 (m, 9H), 1.79–1.72 (m, 1H). ^13^C-NMR (100 MHz, CDCl_3_): δ/ppm = 170.71, 170.12, 169.93, 97.27, 69.22, 68.83, 68.18, 66.55, 62.35, 50.44, 34.82, 20.96, 20.77, 20.74. HRMS (*m*/*z*): calcd for C_14_H_21_N_3_O_8_ [M + Na]^+^: 382.1221, found: 382.1211.

#### 3.2.4. Preparation of (2*R*,3*S*,4*R*)-2-(acetoxymethyl)-6-(2-aminoethoxy) tetrahydro-2*H*-pyran-3,4-diyl Diacetate (Compound **5**)

Lindlar catalyst (0.6 g, 0.3 mmol, 10.0% purity) and p-TSA (0.19 g, 1.1 mmol) were added to a solution of compound **4** (3.0 g, 9.2 mmol) in EtOH (70 mL). The mixture was stirred under H_2_ (15 psi) at 25 °C for 16 h. The mixture was diluted with ethanol and then filtered, and the filtrate was concentrated under reduced pressure to obtain (2*R*,3*S*,4*R*)-2-(acetoxymethyl)-6-(2-aminoethoxy) tetrahydro-2*H*-pyran-3,4-diyl diacetate (3.82 g, crude) as an off-white foam [28]. ^1^H-NMR (400 MHz, CDCl_3_): δ/ppm = 7.71 (d, *J* = 8.0 Hz, 1H), 7.11 (d, *J* = 8.0 Hz, 1H), 6.38 (brs, 2H), 5.28–5.19 (m, 1H), 4.95–4.86 (m, 2H), 4.22 (m, 1H), 3.96–3.89 (m, 1H), 3.77–3.66 (m, 2H), 3.53–3.44 (m, 1H), 3.12–3.01 (m, 2H), 2.34 (s, 3H), 2.21 (m, 1H), 2.10–1.92 (m, 9H), 1.69–1.62 (m, 1H). ^13^C-NMR (100 MHz, CDCl_3_): δ/ppm = 170.77, 170.29, 169.81, 97.39, 69.07, 68.83, 68.18, 66.55, 62.35, 50.44, 34.82, 20.96, 20.77, 20.74. HRMS (*m*/*z*): calcd for C_14_H_24_NO_8_ [M + H]^+^: 334.1496, found: 334.1506.

#### 3.2.5. Preparation of N2-(4-(((2-amino-4-hydroxypteridin-6-yl) methyl) Amino) benzoyl)-N5-(2-(((2*S*,4*R*,5*S*,6*R*)-4,5-diacetoxy-6-(acetoxymethyl) tetrahydro-2*H*-pyran-2-yl) oxy) ethyl)-l-glutamine (Compound **6**)

Compound **5** (1.75 g, 3.5 mmol) and DCC (1.5 g, 7.2 mmol) were added to a solution of folic acid (1.25 g, 2.88 mmol) in DMSO (100 mL) and pyridine (20 g, 0.25 mol, 21 mL). The mixture was stirred at 25 °C for 16 h in darkness. Then the reaction mixture was concentrated under reduced pressure to remove most of the pyridine. The residue was washed with MTBE (500 mL) to obtain the crude product compound **6** (4.4 g, crude) as a red solid. ^1^H-NMR (400 MHz, DMSO): δ/ppm = 11.44 (s, 1H), 8.64 (s, 1H), 7.95 (m, 1H), 7.66 (d, *J* = 8 Hz,1H), 7.19 (s, 1H), 7.13 (s, 1H), 7.07 (s, 1H), 6.93(s, 1H), 6.63 (t, *J* = 8 Hz, 1H), 5.14 (m, 1H), 4.93 (s, 1H), 4.82 (m, 1H), 4.48 (s, 1H), 4.35 (m, 1H), 4.16 (m, 1H), 3.97–3.91 (m, 2H), 3.56 (m, 1H), 3.42 (m, 1H), 3.28 (m, 1H), 2.54 (s, 1H), 2.26(m, 1H), 2.08 (m, 1H), 2.01 (s, 6H), 1.98 (s, 3H). ^13^C-NMR (100 MHz, DMSO): δ/ppm = 174.52, 172.38, 170.58, 170.15, 169.87, 166.68, 158.13, 151.23, 148.99, 129.51, 128.41, 121.82, 118.56, 117.06, 112.86, 111.59, 96.66, 69.43, 68.98, 67.76, 66.27, 63.89, 62.48, 53.23, 46.38, 42.58, 38.93, 34.82, 30.98, 27.45, 21.12, 20.97, 20.91. HRMS (m/z): calcd for C_33_H_40_N_8_O_13_ [M + H]^+^: 757.2788, found:757.2799.

#### 3.2.6. Preparation of N2-(4-(((2-amino-4-hydroxypteridin-6-yl) methyl) amino) benzoyl)-N5-(2-(((2*S*,4*R*,5*S*,6*R*)-4,5-dihydroxy-6-(hydroxymethyl) tetrahydro-2*H*-pyran-2-yl) oxy) ethyl)-l-glutamine (Target Compound)

To a solution of compound **6** (3 g, 4 mmol) in MeOH (61 mL), NaOMe (6.23 g, 28.8 mmol, 25.0% purity of MeOH) was added dropwise and stirred at 0 °C. The reaction mixture was then stirred at 25 °C for 1 h, acidified with acidic resin until pH = 6 was reached, and filtered. The filtrate was concentrated under reduced pressure to yield a crude product. The crude product was purified with a prep-HPLC (column: Agela Durashell (Bonna-Agela, Tianjin, China) 10 µm, 250 × 50 mm; mobile phase: [water (10 mM NH_4_HCO_3_) -ACN]; B %: 0–15.0%, 20 min). After prep-HPLC purification, the eluent was concentrated or evaporated to remove organic solvents. The residual aqueous solution was lyophilized to obtain the product. ^1^H-NMR (400 MHz, CDCl_3_): δ/ppm = 8.97 (s, 1H), 8.58 (d, *J* = 20 Hz, 2H), 7.95 (d, *J* = 10.6 Hz, 1H), 7.85 (s, 1H), 7.68–7.46 (m, 4H), 6.90 (s, 1H), 6.78 (s, 1H), 6.61 (dd, *J* = 19.0, 8.0 Hz, 2H), 4.78 (s, 1H), 4.46 (s, 1H), 4.34 (s, 1H), 4.20 (s, 1H), 4.06 (s, 1H), 3.64–3.21 (m, 11H), 3.02–2.98 (m,1H), 2.19–2.11 (m, 2H), 1.98 (s, 1H), 1.39 (t, *J* = 12.4 Hz, 1H). ^13^C-NMR (100 MHz, DMSO): δ/ppm = 172.81, 166.64, 162.19, 156.88, 155.29, 151.01, 148.83, 129.48, 128.91, 128.41, 121.99, 111.81, 97.19, 73.62, 72.21, 68.38, 65.73, 65.52, 61.47, 54.43, 46.43, 38.91, 38.31, 33.01, 32.69, 29.08, 27.73. HRMS (*m*/*z*): calcd for C_27_H_34_N_8_O_10_ [M + H]^+^: 631.2471, found: 631.2473.

### 3.3. Characterization of Nano-Properties

The nano-properties of FA-2-DG were evaluated by TEM, AFM imaging and DLS measurements.

#### 3.3.1. Transmission Electron Microscopy

The TEM images were recorded on a JEM 2010 microscope (Jeol Ltd., Tokyo, Japan). Briefly, a drop of the sample was placed on a copper grid coated with a carbon film and was allowed to dry for 5 min. In a random region, the features and sizes of the nano-particles were identified by examination of >100 species. An imaging plate with an energy window of 20 eV (Bioscan Camera Model 792; Gatan, Pleasanton, CA, USA) was used to record 6000–40,000× digitally enlarged images [29].

#### 3.3.2. Atomic Force Microscopy

The AFM measurements were recorded on a Multimode 8 instrument (BRUKER, Santa Barbara, CA, USA) in contact mode, and the sample was prepared as a drop of the solution of FA-2-DG at a concentration of 1.6 × 10^−2^ µM placed on a smooth mica plate [30].

#### 3.3.3. Particle Size

A Zetasizer Nano ZS (Malvern, UK) instrument was used to measure the particle size of FA-2-DG at the concentration of 1.6 × 10^−3^ mM [31].

### 3.4. Biological in Vitro Assays

#### 3.4.1. Cell Culture

The MCF-7 cells were grown in Dulbecco’s Modified Eagle’s medium (DMEM, Gibco; Thermo Fisher Scientific, Inc.) supplemented with 100 μg/mL streptomycin, penicillin (Sigma-Aldrich; Merck KGaA, Darmstadt, Germany) and 10% (*v*/*v*) FBS (Gibco; Thermo Fisher Scientific, Inc.). The cells were cultured at 37 °C and 5% CO_2_ in a humidified environment [32].

The SKOV-3 cells were cultured in minimum essential medium (MEM) containing 5% fetal calf serum, l-glutamine (2 mM) and a combination of streptomycin 0.1 mg/mL and penicillin 100 U/mL. The cells were incubated at 37 °C in a humidified air atmosphere containing 5% CO_2_ [33].

#### 3.4.2. Cell Viability Assays

The cell viability of the compounds to the MCF-7 (human breast cancer) and SKOV-3 (human ovarian cancer) cells was determined by MTT assays. Cisplatin was a positive control and blank DMSO was a negative control. Briefly, the cells were cultured in 96-well plates at a suitable density (3000 cells per well) for 24 h before treatment. The solutions for each treatment group were then added. After incubation for the given period, the culture medium was replaced by 100 μL of MTT at 0.5 mg/mL, and the cells were incubated for a further 4 h. The medium solution was removed, and an aliquot of 100 μL DMSO was added. The cytotoxicity against the tested cells was measured at 570 nm with an auto-microplate reader (Infinite M200, Tecan, Männedorf, Switzerland) after the crystals in the wells were dissolved by gentle shaking [34].

#### 3.4.3. Flow Cytometric Analysis of the Cell Cycle and Measurement of the Apoptotic Ratio

Tumor cells were plated in six-well plates at 3 × 10^5^ cells per well. After 24 h incubation, cells in each group were treated for 24 h to analyze the cell cycle and for 48 h to measure cell apoptosis. Cells incubated in medium without treatment were used as controls. For cell cycle analysis [35], the cells were harvested with trypsin, washed with cold PBS and fixed with 70% ethanol at 4 °C overnight. Before analysis, the cells were washed with PBS and stained with 50 μg/mL PI and 50 μg/mL RNase at 37 °C for 15 min. Finally, samples were immediately measured with a CytoFLEX cytometer (Beckman Coulter, Pasadena, CA, USA). For cell apoptotic measurements [36], the cells in different treated groups were harvested, washed with PBS and then treated with trypsin-EDTA solution to detach the cells. The suspended cells were centrifuged at 200× *g* for 10 min. After being washed twice with cold PBS, cells were collected by centrifugation at 200× *g* for 5 min and stained with FITC-conjugated Annexin V and PI. Then, the samples were immediately measured with a CytoFLEX cytometer (Beckman Coulter, Pasadena CA, USA).

### 3.5. Docking Simulation of FA-2-DG and Folate Receptor

The simulation of FA-2-DG docking onto the folate receptor was performed in Discovery Studio 4.5 (San Diego, CA, USA). The 3-D structure of the folate receptor was from the crystal structure of human FRα in complex with folic acid (PDB ID: 4LRH), as a docking pocket, and the binding site of folic acid to the folate receptor was predefined [37]. To prepare FA-2-DG for the docking simulation, we first converted the 2-D structure to a 3-D structure, and energy minimization was performed. The prediction of FA-2-DG docking onto the folate receptor was analyzed with CDOCKER on the basis of the CHARMM force field [38,39].

### 3.6. Statistical Analysis

All results are shown as means and standard deviations calculated from the measurements, and all experiments were carried out in three replicates to obtain these measurements. Data were analyzed via Student’s *t*-test (comparing two samples) or ANOVA (comparing more than two samples), with multiple pairwise comparisons being carried out using Duncan’s multiple range test. The means were considered significantly different if * *p* < 0.05, ** *p* < 0.01 or *** *p* < 0.001.

## 4. Conclusions

In this work, folic acid-modified 2-DG (FA-2-DG) was synthesized, and its targeting ability was evaluated through a computational study. FA-2-DG exhibited self-assembly ability and formed nano-particles in water. It inhibited cell viability by disturbing glycolysis and blocking the cell cycle, and it induced cell apoptosis. A computational study showed that FA-2-DG can dock onto the folate receptor in the same manner as folic acid. All the results obtained from this study confirmed that FA-2-DG, as a nano-scale pharmacosome, is a candidate for combination targeting anti-tumor therapy.

## References

[1] Gapstur S.M., Gann P.H., Lowe W., Liu K., Colangelo L., Dyer A. (2000). Abnormal glucose metabolism and pancreatic cancer mortality. JAMA.

[2] Warmoes M.O., Locasale J.W. (2014). Heterogeneity of glycolysis in cancers and therapeutic opportunities. Biochem. Pharmacol..

[3] Pelicano H., Martin D.S., Xu R.H., Huang P. (2006). Glycolysis inhibition for anticancer treatment. Oncogene.

[4] Diaz-Ruiz R., Rigoulet M., Devin A. (2011). The warburg and crabtree effects: On the origin of cancer cell energy metabolism and of yeast glucose repression. Biochim. Biophys. Acta.

[5] Zheng J.I. (2012). Energy metabolism of cancer: Glycolysis versus oxidative phosphorylation (Review). Oncol. Lett..

[6] Gatenby R.A., Gillies R.J. (2004). Why do cancers have high aerobic glycolysis?. Nat. Rev. Cancer.

[7] O’Byrne K.J., Baird A., Kilmartin L., Leonard J., Sacevich C., Gray S.G. (2011). Epigenetic regulation of glucose transporters in non-small cell lung cancer. Cancers.

[8] Kondoh H., Lleonart M.E., Gil J., Wang J., Degan P., Peters G., Martinez D., Carnero A., Beach D. (2005). Glycolytic enzymes can modulate cellular life span. Cancer Res..

[9] Sottnik J.L., Lori J.C., Rose B.J., Thamm D.H. (2011). Glycolysis inhibition by 2-deoxy-d-glucose reverts the metastatic phenotype in vitro and in vivo. Clin. Exp. Metastasis.

[10] Zhang D., Li J., Wang F., Hu J., Wang S., Sun Y. (2014). 2-Deoxy-D-glucose targeting of glucose metabolism in cancer cells as a potential therapy. Cancer Lett..

[11] Maher J.C., Savaraj N., Priebe W., Liu H., Lampidis T.J. (2005). Differential sensitivity to 2-deoxy-d-glucose between two pancreatic cell lines correlates with glut-1 expression. Pancreas.

[12] Urakami K., ZangiacomiI V., Yamaguchi K., Kusuhara M. (2013). Impact of 2-deoxy-D-glucose on the target metabolome profile of a human endometrial cancer cell line. Biomed. Res..

[13] Giammarioli A.M., Gambardella L., Barbati C., Pietraforte D., Tinari A., Alberton M., Gnessi L., Griffin R.J., Minetti M., Malorni W. (2012). Differential effects of the glycolysis inhibitor 2-deoxy-D-glucose on the activity of pro-apoptotic agents in metastatic melanoma cells, and induction of a cytoprotective autophagic response. Int. J. Cancer.

[14] Wilkes G.M. (2018). Targeted therapy: Attacking cancer with molecular and immunological targeted agents. Asia Pac. J. Oncol. Nurs..

[15] Arap M.A., Arap W., Lahdenranta J., Mintz P.J., Hajitou A., Sarkis Á.S., Pasqualini R. (2004). Cell surface expression of the stress response chaperone GRP78 enables tumor targeting by circulating ligands. Cancer Cell.

[16] O’Shannessy D.J., Somers E.B., Wang L., Wang H., Hsu R. (2015). Expression of folate receptors alpha and beta in normal and cancerous gynecologic tissues: Correlation of expression of the beta isoform with macrophage markers. J. Ovarian Res..

[17] Kalli K.R., Oberg A.L., Keeney G.L., Christianson T.J.H., Low P.S., Knutson K.L., Hartmann L.C. (2008). Folate receptor alpha as a tumor target in epithelial ovarian cancer. Gynecol. Oncol..

[18] Van Dam G.M., Themelis G., Crane L.M.A., Harlaar N.J., Pleijhuis R.G., Kelder W., Sarantopoulos A., de Jong J.S., Arts H.J.G., van der Zee A.G.J. (2011). Intraoperative tumor-specific fluorescence imaging in ovarian cancer by folate receptor-α targeting: First in-human results. Nat. Med..

[19] Bwatanglang I.B., Mohammad F., Yusof N.A., Abdullah J., Alitheen N.B., Hussein M.Z., Abu N., Mohammed N.E., Nordin N., Zamberi N.R. (2016). In vivo tumor targeting and anti-tumor effects of 5-fluororacil loaded, folic acid targeted quantum dot system. J. Colloid Interface Sci..

[20] Li X., Szewczuk M., Malardier-Jugroot C. (2016). Folic acid-conjugated amphiphilic alternating copolymer as a new active tumor targeting drug delivery platform. Drug Des. Dev. Ther..

[21] Hao Y., Wang L., Zhang B., Zhao H., Niu M., Hu Y., Zheng C., Zhang H., Chang J., Zhang Z. (2016). Multifunctional nanosheets based on folic acid modified manganese oxide for tumor-targeting theranostic application. Nanotechnology.

[22] Zhang M., Guo R., Wang Y., Cao X., Shen M., Shi X. (2011). Multifunctional dendrimer/combretastatin A4 inclusion complexes enable in vitro targeted cancer therapy. Int. J. Nanomed..

[23] Moghimipour E., Rezaei M., Ramezani Z., Kouchak M., Amini M., Angali K.A., Dorkoosh F.A., Handali S. (2018). Folic acid-modified liposomal drug delivery strategy for tumor targeting of 5-fluorouracil. Eur. J. Pharm. Sci..

[24] Zhao C., Cheng R., Yang Z., Tian Z. (2018). Nanotechnology for cancer therapy based on chemotherapy. Molecules.

[25] Wang Y., Yang P., Zhao X., Gao D., Sun N., Tian Z., Ma T., Yang Z. (2018). Multifunctional cargo-free nanomedicine for cancer therapy. Int. J. Mol. Sci..

[26] Amit K., Vipin K., Dere R.T., Schmidt R.R. (2011). Glycoside bond formation via acid-base catalysis. Org. Lett..

[27] Zhu W., Ma D. (2004). Synthesis of aryl azides and vinyl azides via proline-promoted CuI-catalyzed coupling reactions. Chem. Commun..

[28] Reddy P.G., Pratap T.V., Kumar G.D.K., Mohanty S.K., Baskaran S. (2003). The Lindlar Catalyst Revitalized: A Highly Chemoselective Method for the Direct Conversion of Azides to N-(tert-Butoxycarbonyl)amines. Cheminform.

[29] Toro-Cordova A., Flores-Cruz M., Santoyo-Salazar J., Carrillo-Nava E., Jurado R., Figueroa-Rodriguez P., Lopez-Sanchez P., Medina L., Garcia-Lopez P. (2018). Liposomes loaded with cisplatin and magnetic nanoparticles: Physicochemical characterization, pharmacokinetics, and In-Vitro efficacy. Molecules.

[30] Valencia C., Valencia C., Zuluaga F., Valencia M., Mina J., Grande-Tovar C. (2018). Synthesis and application of scaffolds of chitosan-graphene oxide by the freeze-drying method for tissue regeneration. Molecules.

[31] Li H., Yang C., Chen C., Ren F., Li Y., Mu Z., Wang P. (2018). The use of trisodium citrate to improve the textural properties of acid-Induced, transglutaminase-treated micellar casein gels. Molecules.

[32] Turan-Zitouni G., Yurttaş L., Tabbi A., Akalın Çiftçi G., Temel H., Kaplancıklı Z. (2018). New thiazoline-tetralin derivatives and biological activity evaluation. Molecules.

[33] Fan D., He T., Wang Y., Kong G., Jiang T., Zhou D. (2011). Production, preliminary characterization and antitumor activity (SKOV-3 cell lines) in vitro of glycans from green tea. Carbohydr. Polym..

[34] Liu J., Wei T., Zhao J., Huang Y., Deng H., Kumar A., Wang C., Liang Z., Ma X., Liang X.J. (2016). Multifunctional aptamer-based nanoparticles for targeted drug delivery to circumvent cancer resistance. Biomaterials.

[35] Wang R., Zhang Q., Peng X., Zhou C., Zhong Y., Chen X., Qiu Y., Jin M., Gong M., Kong D. (2016). Stellettin B induces G1 arrest, apoptosis and autophagy in human non-small cell lung cancer A549 cells via blocking PI3K/Akt/mTOR pathway. Sci. Rep..

[36] Wang Z., Wang Y., Zhu S., Liu Y., Peng X., Zhang S., Zhang Z., Qiu Y., Jin M., Wang R. (2018). DT-13 Inhibits proliferation and Metastasis of Human Prostate Cancer Cells Through Blocking PI3K/Akt Pathway. Front. Pharmacol..

[37] Chen C., Ke J., Zhou X.E., Yi W., Brunzelle J.S., Li J., Yong E., Xu H.E., Melcher K. (2013). Structural basis for molecular recognition of folic acid by folate receptors. Nature.

[38] Jin W., Ma Y., Li W., Li H., Wang R. (2018). Scaffold-based novel SHP2 allosteric inhibitors design using Receptor-Ligand pharmacophore model, virtual screening and molecular dynamics. Comput. Biol. Chem..

[39] Regulski M., Piotrowska-Kempisty H., Prukała W., Dutkiewicz Z., Regulska K., Stanisz B., Murias M. (2018). Synthesis, in vitro and in silico evaluation of novel trans -stilbene analogues as potential COX-2 inhibitors. Bioorg. Med. Chem..

